# Purification of Pharmaceutical Solvents by Pervaporation through Hybrid Silica Membranes

**DOI:** 10.3390/membranes9070076

**Published:** 2019-07-01

**Authors:** Thomas La Rocca, Emilie Carretier, Didier Dhaler, Eric Louradour, Thien Truong, Philippe Moulin

**Affiliations:** 1 Aix-Marseille Université, CNRS Centrale Marseille, M2P2 UMR 7340, Equipe Procédés Membranaires (EPM), Europôle de l’Arbois, BP80, Pavillon Laennec, Hall C, 13545 Aix-en-Provence, CEDEX, France; 2 Céramiques Techniques Industrielles (CTI), 382 Avenue du Moulinas, 30340 Salindres, France

**Keywords:** solvents purification, pervaporation, hybrid silica membrane, coupling effect

## Abstract

Solvents purification mainly used in pharmaceutical field such as acetone and methyl ethyl ketone (MEK) were performed through hybrid silica membranes and from binary and multi-components mixtures. Two hybrid silica membranes—zirconia doped bis(triethoxysilyl)methane and bis(triethoxysilyl)ethane (BTESE)—were studied. Flux, permeance, and separation factor were evaluated depending on temperature, composition, and number of organic compounds in the feed. Dehydration tests of acetone were operated at 30 and 45 °C following by acetone and MEK purification at 50 °C from multi-components hydro-organic mixtures where hydrophilic compounds (water, methanol) but also hydrophobic (dichloromethane (DCM) and/or toluene) were present. Results showed that the presence of Zr nanoparticles affected flux and improved selectivity in the case of dehydration. Experiments related to acetone and MEK purification, revealed a mass transfer alteration and a decrease of performance, from 99 to 97 wt% and from 98 to 95 wt% respectively, when the number of compounds in the initial feed grown up and more precisely, in the presence of DCM and toluene thus highlighting a possible coupling effect.

## 1. Introduction

Organic compounds are mostly used as solvents in industrial fields such petrochemical, food industry, pharmaceutical, or water treatment due their physicochemical properties more suitable than water [1,2]. In the pharmaceutical sector notably and related to this work, they are used without chemical modification for the excipient and active ingredient fabrication which imposes no residue of water. On the global scale, the annual consumption of solvents is in continuous growth with an estimated increase of 18 to 23 megatons between 2013 and 2020 [2] which involves the use of effective processes to purify them. However, some of them are carcinogenic, mutagenic, and toxic for reproduction (CMR) substances and cause severe damages to the environment with for example, the smog formation in the troposphere. That is why, it is necessary to treat them in order to reduce their atmospheric emissions [3,4].

Regenerative processes such as distillation and liquid-liquid extraction [5,6] are a good alternative to destructive processes because they offer both environmental and economic benefits. Indeed, they limit the extraction of raw material and reduce the financial cost for industrials related to news solvents. Among them, pervaporation aims to extract minority organic compounds (less than 10 wt%) from a liquid mixture through a non-porous membrane [7,8]. This process requires less energy than conventional processes such as distillation that consumes up to 60% more energy [9], and can also separate azeotropic mixtures because the separation is based on solubility and diffusivity differences of compounds in the membrane [10]. Pervaporation is mainly used for alcohol dehydration (ethanol, isopropanol) [11,12], VOC extraction [13,14,15] such as chlorinated compounds [16,17], toluene [18], or benzene [19], and lastly for the organic/organic separation (isomers, benzene/cyclohexane) [20]. More precisely, experiments pervaporation with acetone are mainly applied for its dehydration [21,22,23], acetone-ethanol-butanol (ABE) recovery for fermentation broth [24,25], and more rarely for acetone-organic separation such as acetone/n-hexane [26]. Similarly, pervaporation tests for MEK are primarily focused on its extraction from water [27,28]. Thus, no experiments focused on acetone or MEK purification by pervaporation from hydro-organic multicomponent mixtures were listed more recently. However, contaminated industrial mixtures often contains several organic pollutants.

In this work, dehydration and purification of acetone and MEK (90 wt %) were performed through two hybrid silica membranes with different precursors, from multi-components mixtures at several temperature. In the first time pure water, ethanol, acetone, and MEK permeations were done at 30 or 45 °C following by the acetone dehydration. Flux, permeance and separation factor were compared between hybrid silica membranes and according to temperature. Then, ternary acetone-water-methanol and acetone-water-DCM, quaternary acetone-water-methanol-DCM then MEK-water-methanol-DCM and lastly MEK-water-methanol-DCM-acetone-toluene were studied at 50 °C. The mass fraction of water was between 8 to 2 wt% and the other organic compound showed a mass fraction of 2 wt%. These temperatures were chosen in order to make a specific comparison with others membranes founded in the literature where the temperature range is usually between 20 to 70 °C [13]. Hybrid silica membranes were developed by ECN (Netherland) and show a hybrid structure. They mostly applied for alcohol dehydration and present a very good thermal resistance up to 150 and 190 °C in the long and short term respectively [29,30]. The effects of temperature and concentration/number of organic species in the feed on purification performances were studied.

## 2. Theoretical Aspects

Pervaporation is suitable for the extraction of minority compounds with a mass (or molar) fraction which does not usually exceed 10% unless a large membrane surface is used [14]. In this process, a partial vaporization of compound in the membrane is occurred to form a vapor permeate at the downstream side. This change in state occurs thanks to a partial pressure gradient upstream and downstream of the membrane. The solution-diffusion model [31] is often applied to characterize the mass transfer. This model, suited for pervaporation, supposes the existence of vapor-liquid equilibrium (VLE) at the liquid feed-membrane interface. Transmembrane molar flux for a compound *i* is expressed such as:

(1) $$J_{i}=\;\frac{P_{i}^{G}}{\delta }\;\left(\gamma_{i,f}^{L}\;x_{i,f}^{L}\;p_{i,f}^{sat}-\;p_{i}^{p}\right)$$

where $J_{i}$ is the molar flux of compound *i* (mol·m^−2^·s^−1^), $P_{i}^{G}$ is the gas (G) permeability of the membrane (mol·m^−2^·s^−1^.m·Pa^−1^), δ is the membrane thickness (m), $\gamma_{i,f}^{L}$ and $x_{i,f}^{L}$ are the activity coefficient (-) and the molar fraction (-) in the liquid (L) feed (f), $p_{i,f}^{sat}$ is the vapor pressure of pure compound *i* in the feed (Pa), $p_{i}^{p}$ is the partial pressure of i (Pa) at permeate side (p).

Baker et al. [32] suggested the permeance term $\frac{P_{i}^{G}}{\delta }$ (mol·m^−2^·s^−1^·Pa^−1^) if the membrane thickness is unknown:

(2) $$\frac{\;P_{i}^{G}}{\delta }=\;\frac{J_{i}}{\left(\gamma_{i,f}^{L}\;x_{i,f}^{L}\;p_{i,f}^{sat}-\;p_{i}^{p}\right)}$$

Most of workers report the permeability as Barrer where 1 Barrer = 1 × 10^−^^10^ cm^3^ (STP)·cm^−^^2^·s^−^^1^·cm·cmHg^−^^1^. Similarly, permeance is usually reported as gpu and 1 gpu = 1 × 10^−^^6^ cm^3^ (STP)·cm^−2^·s^−1^·cmHg^−1^. This latter unit was used in this work. The NRTL (Non-Random Two-Liquid) is often applied to determine activity coefficients (γ) in the liquid phase from a binary mixture (compound *i* and *j*) [33]. 

Several operating parameters affects the global performance of process such as temperature, hydrodynamics (Reynolds number), concentration, or number of organic compounds in the feed. Temperature effects can be describe by the Arrhenius equation [34,35]:

(3) $$J_{i}=\;J_{0,i}\;exp\left(\frac{-E_{a,i}}{RT}\right)$$

where $J_{0,i}$ represents the pre-exponential factor of *i* (mol*·*m^−2^*·*h^−1^), $E_{a,i}$ denotes the activation energy of *i* (kJ*·*mol^−1^), R  is the ideal gas constant (kJ*·*mol^−1^*·*K^−1^) and *T* is the temperature (K).

The activation energy is thus a characteristic variable to describe the benefit influence of temperature on flux. Luis et al. [36] suggest that the higher its absolute value is set, and more the effects of temperature are important. For example in the case of dehydration through a silicalite membrane, the activation energy of water is greater than the one of solvent, the increase of temperature improves both flux and selectivity [27]. Organic composition is also an important factor because it can affect the driving force of system. Notably, it may lead to the emergence of coupling effect due to mutual interactions between compounds [37].

## 3. Materials and Methods

### 3.1. Chemical Products and Membrane

All chemical products were purchased in Thermofischer and had a purity of 99.5 wt%. Table 1 lists chemical products used in this work as well as their physicochemical properties such the water/octanol partition coefficient (Log Kow) and kinetic diameter. Distilled water was used.

**Figure 1:**
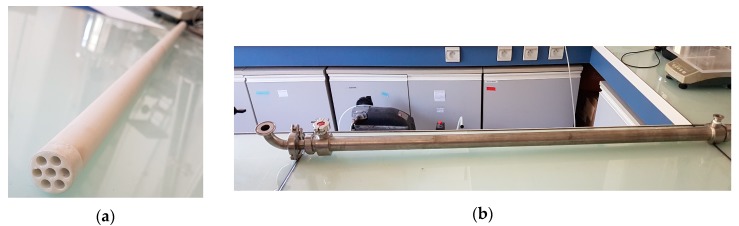
Material membrane used in this study: (**a**) hybrid silica membrane; (**b**) membrane module.

Membranes used for this study, hydrophilic hybrid silica membrane [38,39], were purchased from Céramiques Techniques Industrielles (CTI, Salindres, France). Their structures are composed with a ceramic support and a hybrid organic/inorganic top layer made with hybrid silica. In this case, two membranes with different precursors were tested: A membrane named M1 and made with [bis(triethoxylsil)ethane] (BTESE), and another called M2 made with Zirconia doped BTESM. The two membrane are tube-shaped with seven channels and measures 1.2 m long for an active area of 0.155 m^2^ (Figure 1a,b).

### 3.2. Experimental Set-Up

**Figure 2:**
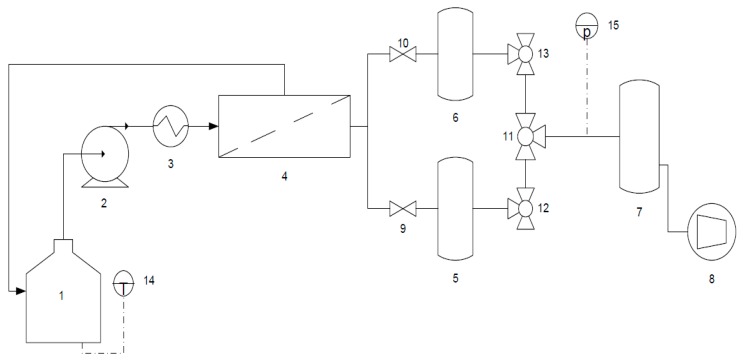
Pervaporation process set-up: (1) Feed tank ; (2) Volumetric pump ; (3) Heat exchanger ; (4) Membrane ; (5,6) Cold traps with liquid nitrogen ; (7) Safety trap ; (8) Vacuum pump ; (9,10) Simple valve ; (11,12,13) 3-way valves ; (14) Temperature sensor (±0.2 °C from −30 to 120 °C) ; (15) Vacuum pressure sensor (<± 10% from 10^−2^ to 20 mbar).

The process flowsheet is shown in Figure 2. The feed is prepared in a 3 L tank which is equipped with a temperature sensor (Hanna Instruments, France). A volumetric pump circulates the liquid towards the membrane with an operating flowrate of 400 L·h^−1^ to ensure a turbulent regime in the membrane and limit the concentration polarization near the membrane wall even if in pervaporation this phenomenon appears no significant. A heat exchanger (Serflam, France) controls the operating temperature. A vacuum pump (ATB, Austria) is used to create the difference of partial pressure between feed and downstream (permeate) side. It allows to keep low pressure of 3 mbar at the permeate side with a variation less than 1 mbar. In order to regularly verify the downstream pressure, a vacuum pressure sensor (Thyracont VD84, Passau, Germany) is included in the permeate circuit. The condensation and recovery of permeate is performed by 1 L cold traps with liquid nitrogen at −196 °C. A 3-way valves operates the switching between two traps and a third trap, called safety trap, runs continuously to avoid the contact between vacuum pump and last uncondensed vapors.

Simultaneously, sampling at the feed/retentate and permeate side was performed regularly. Depending on mixture, two analysis devices were applied: Samples from binary mixtures were quantified by a densimeter (Anton Paar, Graz, Austria) showing a high precision of ±10^−6^ g·cm^−3^ and a gas chromatography was used to analyze samples from multi-components. Purification performances were evaluated calculating the mass flux of permeate from a same cold trap, as follows:

(4) $$J_{p}=\;\frac{m_{p}}{A\;\Delta T}$$

where *J_p_* the mass total flux of permeate (kg·m^−2^·h^−1^), *m_p_* is the mass of permeate collected in the trap for a period of Δ*t* (h), *A* is the membrane area (m^2^).

Separation factor α is then calculated such as:

(5) αi,j= wp,iwp,jwf,iwf,j

where  w represents the mass fraction of species *i* or *j* (%) and permeate and feed (or retentate) side are indicated by subscripts p and f respectively.

**Figure 3:**
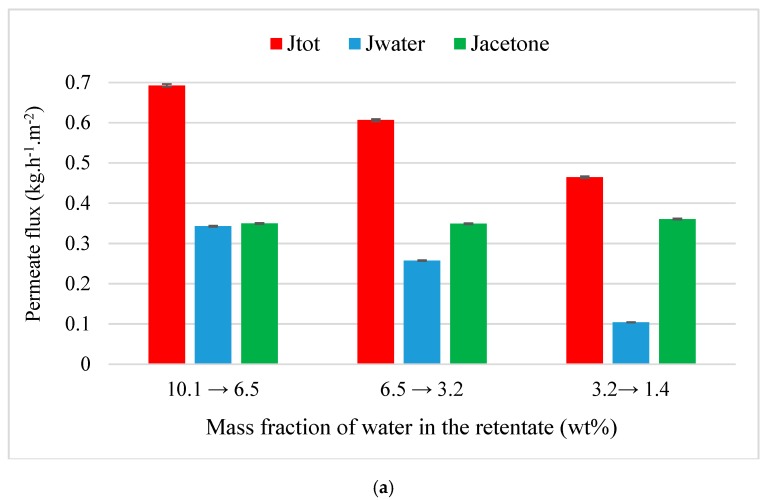

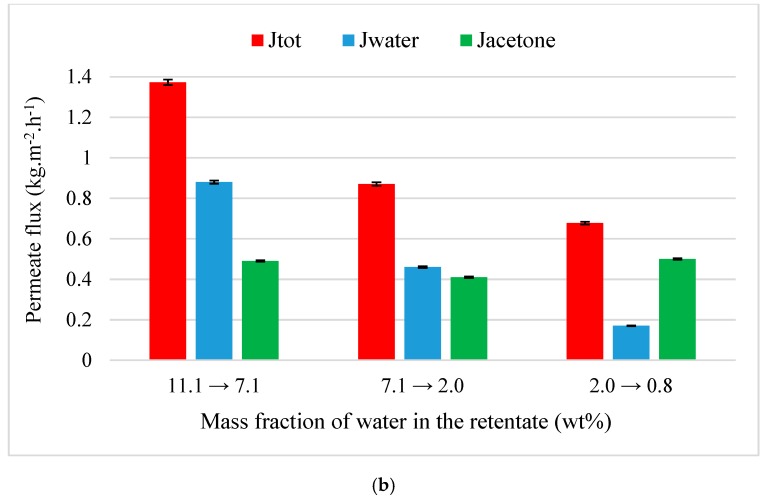
Evolution of flux as the function of the inlet composition from a binary acetone-water mixture (90/10 wt%) through M1 depending of temperature: (**a**) 30 °C; (**b**) 45 °C.

**Figure 4:**
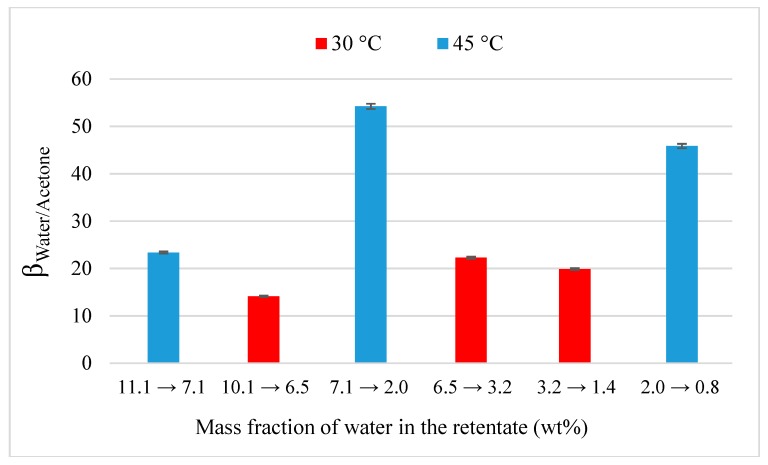
Evolution of separation factor water/acetone as the function of the inlet composition through M1 at 30 and 45 °C.

**Figure 5:**
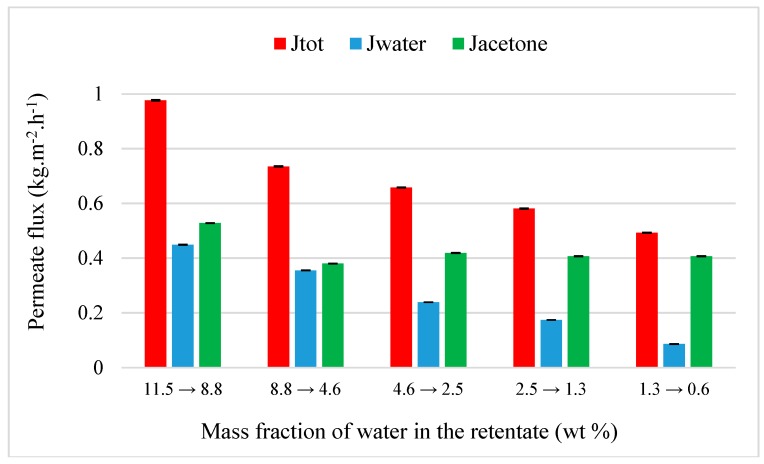
Evolution of flux as the function of the inlet water composition from a binary acetone-water mixture (90/10 wt%) through M2 at 30 °C.

**Figure 6:**
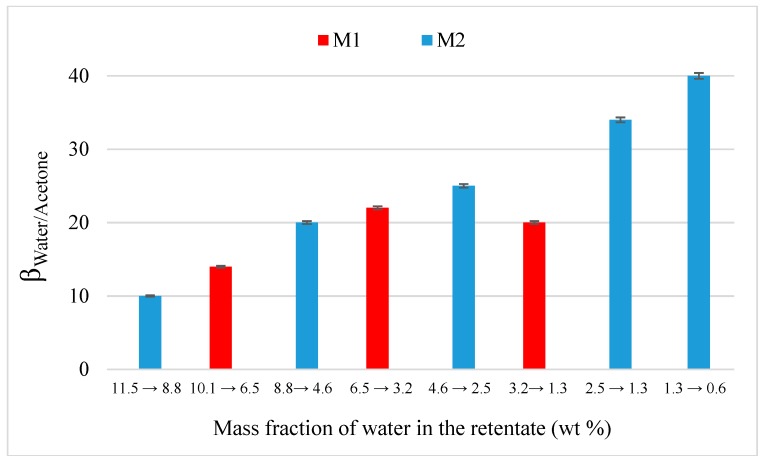
Evolution of separation factor water/acetone as the function of the inlet water composition through M1 and M2 at 30 °C.

**Figure 7:**
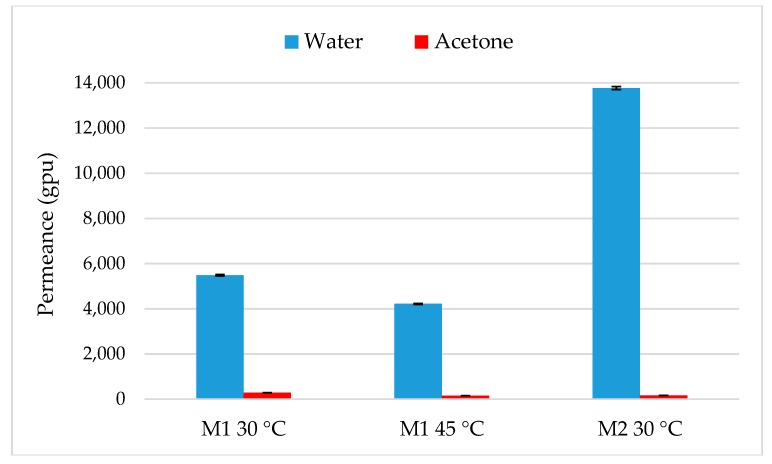
Permeance values for water and acetone depending on temperature and membrane.

**Figure 8:**
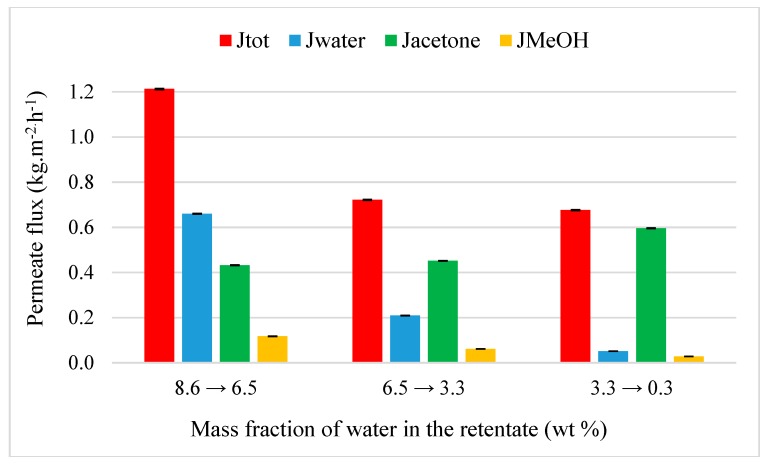
Evolution of flux as the function of the inlet water composition from a ternary acetone-water-MeOH mixture (90.8/7.2/2.0 wt%) through M1 at 50 °C.

**Figure 9:**
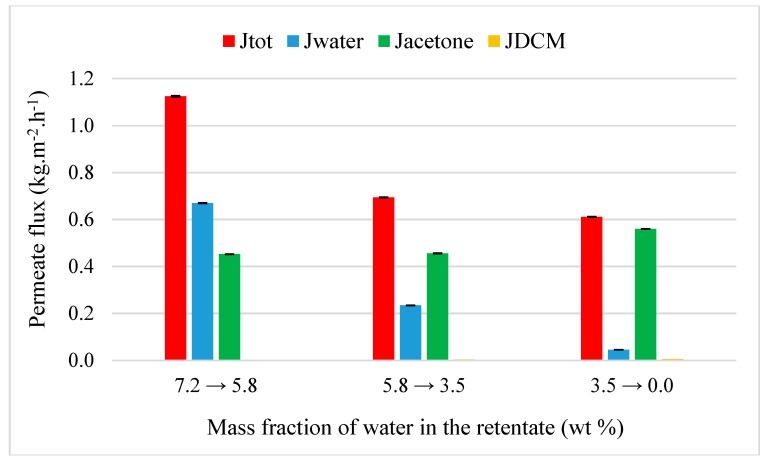
Evolution of flux as the function of the inlet water composition from a ternary acetone-water -DCM mixture (90.8/7.2/2.0 wt%) through M1 at 50 °C.

**Figure 10:**
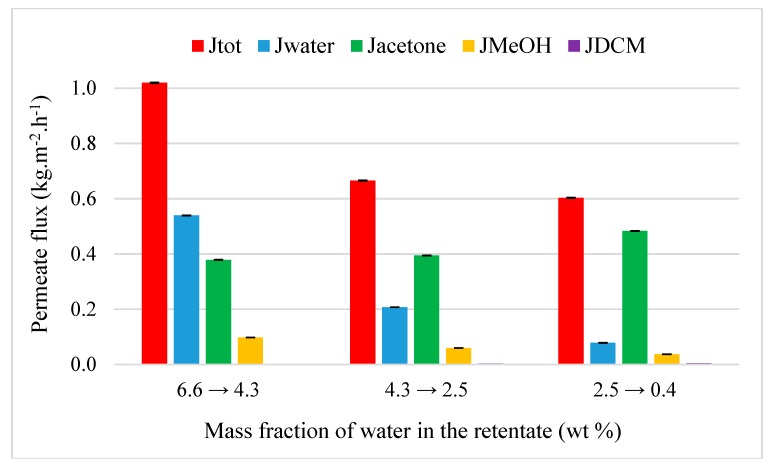
Evolution of flux as the function of the inlet water composition from a quaternary acetone-water–MeOH-DCM mixture (89.4/6.6/2.2/1.8 wt%) through M1 at 50 °C.

**Figure 11:**
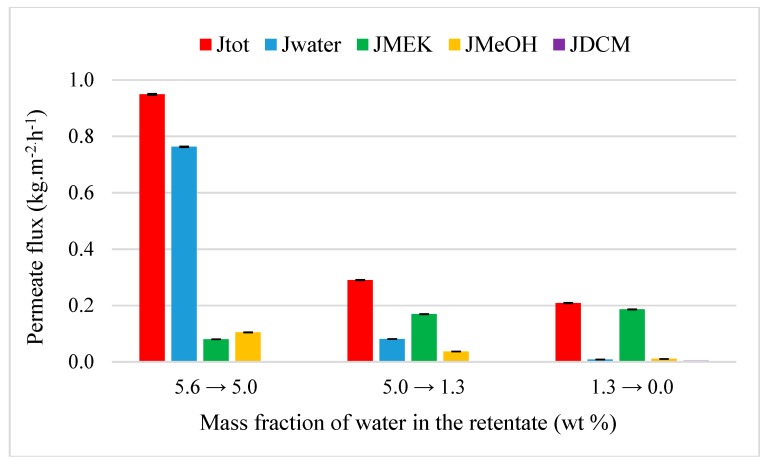
Evolution of flux as the function of the inlet water composition from a quaternary MEK-water–MeOH-DCM mixture (90.6/5.6/1.9/1.9 wt%) through M1 at 50 °C.

**Figure 12:**
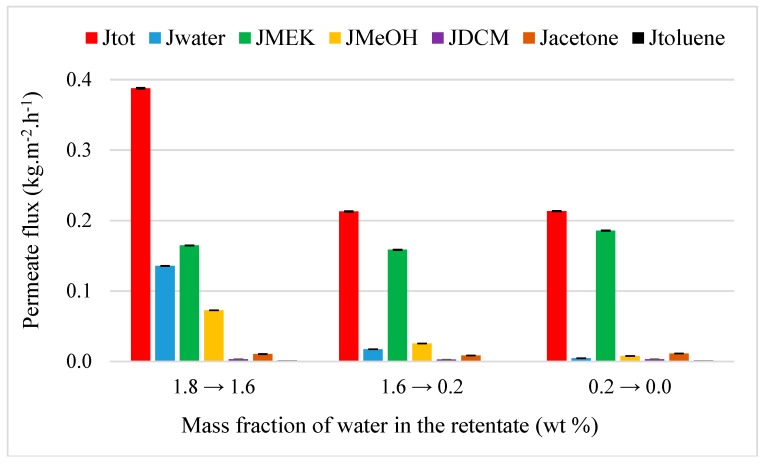
Evolution of flux as the function of the inlet water composition from a multi-component MEK-water–MeOH-DCM-acetone-toluene mixture (90/2.0/2.0/2.0/2.0/2.0 wt%) through M1 at 50 °C.

All pure flux experiments through both M1 and M2 lasted 2 h with a sampling of retentate/permeate and a trap switching every 30 min. For dehydration and purification experiments, initial mass fraction of solvent (acetone or MEK) was equal around 90 wt% and the time experiment chosen in an arbitrary manner according to the purification speed. Therefore, experiments lasted between 2 and 4 h with a sampling and a trap switching between 30 and 60 min. The *x*-axis values given in Figure 3, Figure 4, Figure 5, Figure 6, Figure 7, Figure 8, Figure 9, Figure 10, Figure 11 and Figure 12 (except Figure 7) represents the range of mass fraction of water measured at the beginning and end of the trap switching. Besides, the two membranes were not available at the same time; that is why, organic purification tests were only performed through M1. Operational conditions for each dehydration/purification experiment are summarized in Table 2.

## 4. Results and Discussion

### 4.1. Pure Solvents Permeation

Pure water, ethanol, acetone, and MEK permeation were performed at 30 °C through M1 and M2. For water, experiments were also operating at 45 °C in order to observe effects of temperature on flux and permeance. Results are listed in Table 3.

Results confirm the hydrophilic nature of M1 since the water flux is the most important behind acetone, ethanol then MEK flux. Water flux is also the greatest for M2. The high degree of hydrophilicity and the small kinetic diameter of water (Table 1) promote its transfer in the two membranes. At the opposite, MEK presents a higher molecular size and a more hydrophobic nature (Table 1) than others components what slows its permeation, notably during the diffusion step. Furthermore, for a given compound, flux differ from M1 to M2. More precisely, water flux increases from 1.01 to 1.37 kg·h^−1^·m^−2^ whereas ethanol and acetone flux decrease from 0.71 to 0.47 kg·h^−1^·m^−2^ and from 0.76 to 0.49 kg·h^−1^·m^−2^ respectively from M1 to M2. The kind of precursor (BTESE or BTESM) thus affects the pure solvent permeation. The presence of Zr particles in the top layer of M2 could improve the permeation of very hydrophilic compound as water at the expense of less hydrophilic compound as acetone. Therefore, M2 could show better performances of dehydration. The increase of temperature improves the water flux which strongly increases when the temperature raised from 30 to 45 °C. Also, permeance values were calculated from the Equation (2). For each membrane and at 30 °C, the transition between a hydrophilic compound (water) toward a less hydrophilic compounds causes a drop of permeance and water remains the most permeable compound. Notably for M1, a factor 10 is observed in Table 3 between water and ethanol permeance then those of ethanol and acetone. This significant difference reflects the influence of driving force and more precisely the partial pressure of compound on the permeation (Table 1). M2 shows a higher permeance for water than M1 as observed for pure flux. On the contrary, a higher permeance for ethanol and acetone is visible with M1. Furthermore, an increase of water permeance is observed when the temperature rises from 30 to 45 °C. At 40 °C, Jin et al. [40] find a water and ethanol permeance through a hybrid membrane with metal organic framework (MOF-CAU-10-H) of 5229 and 299 gpu, respectively. These values are in agreement with the M1, M2 values but the hybrid structure of M1 and M2 made with BTESE or BTESM + Zr improves the productivity and thus, could provide better performances of dehydration.

### 4.2. Acetone Dehydration

In the first time, flux and separation factor during acetone dehydration (90 wt%) were evaluated at 30 and 45 °C through M1 (Figure 3a,b). For each temperature, total and water flux decrease when the mass fraction of water in the retentate decreases whereas acetone flux is relatively constant to 0.35 and 0.47 kg·h^−1^·m^−2^ at 30 and 45 °C, respectively. The increase of temperature promotes initial total flux which rises from 0.69 to 1.37 kg·h^−1^·m^−2^. Similarly, water flux increases to 0.34 to 0.88 kg·h^−1^·m^−2^ when temperature rises from 30 to 45 °C. In the all cases, initial flux of water is less than that of its pure flux (Table 3). This can be achieved by the drop of partial pressure of water in the binary mixture. Globally, dehydration performance is improved when the increase of temperature and the decrease of water flux is faster at 45 °C than 30 °C. This is confirmed by the Figure 4 that represents the evolution of separation factor water/acetone at 30 and 45 °C.

Greater values of selectivity are observed when the temperature rises leading so to higher dehydration performances. The rigid structure of two hybrid silica membranes, based on ceramic support, prevents the swelling phenomena which leads to an increase of separation factor. On the contrary, this swelling affects hydrophilic membranes more flexible as PEBA [12] or PEBAX/PVDF [41] membranes, causing an increase of free volume in their structure and a permeation more important for the both compounds at the expense of selectivity. Besides, in accordance with Arrhenius’ equation, the higher values of activation energy for water (between 34 and 64 kJ·mol^−1^) compared to those for acetone (22 kJ·mol^−1^) in silicalite membrane could be improve the selectivity between the two compounds when the temperature increases. At the end of dehydration, an opposite evolution is observed where separation factor decreases down to 20 and 46 at 30 and 45 °C, respectively, when water composition reach for 0 wt%. Therefore, the kind of precursor (BTESE or BTESM) could lead to a different behavior of M1 and M2 against selectivity. The presence of Zr nanoparticles in M2 could improve the selectivity for very low fractions of water.

Evolution of flux through M2 at 30 °C is shown in Figure 5. As observed with M1 (Figure 3a), total and water flux decreases when the water fraction decreases, and the acetone flux remains constant around 0.41 kg·h^−1^·m^−2^ which is consistent with its pure flux measured through the same membrane at 30 °C (Table 3, 0.50 kg·h^−1^·m^−2^). Compared to values of flux through M1, global permeation is higher through M2 where the total and water flux decreases from 0.98 to 0.49 kg·h^−1^·m^−2^ and from 0.45 to 0.08 kg·h^−1^·m^−2^ respectively. A similar performance for M2 is observed with a decrease of mass fraction of water from 11.5 to 0.6 wt%.

The difference between membranes is more visible on separation factor (Figure 6). For M2, separation factor continuously increases from 10 to 40 when the water fraction decreases from 11.5 to 0.6 wt%. M2 is thus more selective than M1 and improves dehydration performances of acetone which confirms results from pure solvents permeation. Permeance values, illustrated in Figure 7, were calculated from Equation (2), taking into account the average concentration in the range (*x*-asis) and permeate pressure measured with a same trap. The determination of coefficient activity was done with the NRTL parameters given by Tochigi and al. [42] for a binary acetone-water mixture. A significant drop of water permeance is observed for both membrane with the transition between pure and binary mixture. This drop is more important for M1 with a decrease from 11,950 to 5488 and from 13,019 to 4220 gpu at 30 and 45 °C respectively. In the case of binary mixture, water permeance is thus lower when the temperature increases from 30 to 45 °C. Acetone permeance is slightly less important than its pure value with a drop from 291 to 154 gpu and remains very weak. For M2, a greater water permeance is visible with a lower drop from 16,866 to 13,770 gpu at 30 °C and acetone permeance remains constant around 180 gpu.

Greater water permeances were observed in this study compared to the literature (Table 4) [43]: for an acetone-water (95/5 wt%), lower water permeances were obtained through a poly(vinyl alcohol) with multi-walled carbons nanotubes in a crosslinked chitosan matrix (PVA-MWCNT/CS) membrane with values ranging from 1100 to 300 gpu at 30 and 45 °C respectively. For the authors, the drop of water permeance when the temperature increased can be caused by the increase of free volume in the membrane that results in a higher flux for both water and acetone but in a decrease of selectivity. With the same membrane but without crosslinking, a water permeance of 1910 gpu was estimated. Yeang et al. [43] explained this time the crosslinking leaded to an increase of rigidity of polymer chains, a reduction of free volume and so a lower water permeance. A slight increase of water permeance from 3319 to 4150 gpu at 50 and 60 °C, respectively, was observed by Koch et al. [44] for the acetone dehydration (0.3 kg·kg^−1^ of water) a Pervap^TM^ 1210 (PVA/PAN) membrane. The difference between permeance values of water from the literature are affected by several parameters: (i) The material of membrane that is organic or inorganic structure, (ii) feed temperature, and (iii) the studied range concentration of water. M1 and M2 show a greater permeance for water highlighting their strong potential for dehydration experiments.

### 4.3. Organic Purification from Multi-Components Mixtures

#### 4.3.1. Acetone Purification from Ternary Mixtures

Acetone purification was tested through M1 at 50 °C from two ternary mixtures: Acetone-water-MeOH (89.4/8.6/2.0 wt%) and acetone-water-DCM (90.8/7.2/2.0 wt%). The selection of initial mass fraction for minority organic compounds is based on the fact that mixtures from pharmaceutical industry can contain several residues of chemical products such as 2 wt% for example. Figure 8 represents the evolution of flux as the function of the inlet water composition. For M1 and at the same temperature (30 °C), the addition of methanol caused a weak decrease of global permeation with an initial total flux to 1.21 kg·h^−1^·m^−2^. A diminution of water flux from 0.66 to 0.05 kg.h^-1^.m^-2^ is observed when its fraction in the retentate decreases down to 0.3 wt%. A similar variation is observed for the methanol flux with a decrease from 0.12 to 0.03 kg·h^−1^·m^−2^. M1 is both selective for water (β_water/acetone_ = 34) and methanol (β_MeOH/acetone_ = 8). As the water, methanol diffuses easily trough M1 due its hydrophilicity and its small molecules. In spite of similar of physicochemical properties of with water, the presence of methanol does not affect the water permeation due to its smaller initial mass fraction. Acetone flux slightly increases from 0.43 to 0.60 kg·h^−1^·m^−2^ its mass content in the retentate reaches 99.1 wt% with 0.3 and 0.6 wt% of water and methanol, respectively. The substitution of methanol by DCM leads to a reduction of dehydration performance of acetone (Figure 9). The negligible DCM flux (0.006 kg·h^−1^·m^−2^) shows that M1 is not permeable and selective (β_DCM/acetone_ = 0.4) due to its strong hydrophobicity (Table 1). Global permeation is weakly affected with an initial total flux of 1.13 kg·h^−1^·m^−2^ but this leads to an acetone at 97.8 wt% with 2.2 wt% of DCM in the retentate. The non-permeation of DCM promotes thus the water extraction which is totally recovered in the permeate with a separation factor water/acetone of 8. Water permeation is not influenced by the presence of methanol or DCM.

#### 4.3.2. Acetone and MEK Purification from Quaternary Mixtures

Acetone and MEK purification were evaluated at 50 °C through M1 from respectively acetone-water-MeOH-DCM (89.4/6.6/2.2/1.8 wt%) and MEK-water-MeOH-DCM (90.6/5.6/1.9/1.9 wt%) quaternary mixtures. Figure 10 shows the evolution of flux from acetone-water-MeOH-DCM mixture. 

As observed during the transition of binary to ternary mixture, global permeation decreases with an initial total flux of 1.0 kg·h^−1^·m^−2^ with the addition of a fourth compound. Water and methanol remain the most permeable compounds with a separation factor of 39 and 21, respectively. Also, M1 is not selective for DCM (β_DCM/acetone_ = 0.5). The addition of a fourth minority compound in the feed does not affect or very few the dehydration performance of acetone. When the acetone is replaced by the MEK as a solvent (Figure 11), it can be noticed a slow drop of global permeation with an initial total flux of 0.95 kg·h^−1^·m^−2^ due to the hydrophobic nature of MEK. But a slow better performance of dehydration with 98.3 wt% of purification degree of MEK is obtained. Water is quite extracted (β_water/MEK_ = 4381) in the retentate and the mass fraction of methanol is of 0.1 with a strong separation factor of 69. The main difference between acetone and MEK experiment is the selectivity of membrane for DCM. Contrary to the quaternary mixture with acetone, a low selectivity was observed (β_DCM/MEK_ = 1.2) in presence of MEK. However, this latter is less hydrophobic than DCM (Table 1) but on the contrary, its kinetic diameter is higher. So, the smaller molecules of DCM promotes its permeation and the selectivity of M1 compared to the larger MEK molecules. The result is a slow decrease of mass fraction of DCM in the retentate from 1.9 to 1.6 wt%. This allows to obtain a higher MEK purification.

#### 4.3.3. MEK Purification from MEK-Water-MeOH-DCM-Acetone-Toluene Mixture

MEK purification was performed through M1 at 50 °C from a multi-component mixture. All minority compounds, including water, had a mass fraction about of 2.0 wt%. The evolution of flux is illustrated in Figure 12.

Compared to other mixtures, a significantly decrease of initial total flux is observed, with an evolution from to 0.38 to 0.21 kg·h^−1^·m^−2^ which agrees with the pure flux ok MEK (0.27 kg·h^−1^·m^−2^). Besides, a drop in the performance is also noted with a purification degree for MEK of 94.8 wt% only which can explained by (i) the addition of DCM and toluene (hydrophobic compound) and M1 is not selective for this last compound (β_toluene/MEK_ *=* 0.3), and (ii) a low value of initial water fraction in the feed. As for each addition of compounds, the decrease of performance could suppose the presence of coupling effect when the initial compound in the feed increases as suggested by Lipnizki et al. [37].

## 5. Conclusions

In this work, purification of pharmaceutical solvents such as acetone and MEK were performed through hybrid silica membranes that is either BTESE (M1) or BTESM + Zr (M2) as organic precursor, as the function of the number and type of organic compounds and feed temperature. Experiments for pure solvent permeation validated the influence of precursor on flux. M2 revealed thus a better performance of dehydration of acetone and notably on separation factor. Simultaneously, an improved both flux and separation factor were observed when temperature increased from 30 to 45 °C, thanks to the rigid structure of hybrid silica membranes preventing the swelling phenomena. High water permeances were estimated up to 13,770 gpu for M2 confirming thus their strong potential for the dehydration. This permeance was in agreement with literature but higher for the hybrid silica membranes. In this work the temperature was low, the increase of temperature, if possible, could improve the process efficiency. From multi-components mixtures, a decrease of acetone purification from 99.1 to 97.8 wt% was observed in moving from acetone-water-MeOH to acetone-water-DCM mixture. In the same way, a drop of MEK purification from 98.3 to 94.8 wt% was visible when the initial compound in the feed increases. More precisely, the presence or DCM and more importantly toluene, causes a lower performance due their high hydrophobicity. The loss of performance purification could be explained by the coupling effect which more affects the mass transfer in multi-component mixtures. In all case, the use of an on-line analytical technique [45], like the near-infrared spectroscopy (NIR) could allow a better comprehension of mass transfer. 

## Figures and Tables

**Table 1:** Summary of chemical products and their physicochemical properties.

Compound	Log Kow (-)	Saturated Vapor Pressure at 30 °C (kPa) *	Kinetic Diameter (Å)
Water	-	4.2	2.96
Acetone	−0.24	37.5	4.69
DCM	1.25	70.5	4.60
Ethanol	−0.31	10.4	4.30
MEK	0.29	15.0	5.04
Methanol (MeoH)	−0.77	21.9	3.80
Toluene	2.73	4.9	5.68

* estimated with the Antoine’s equation.

**Table 2:** Summary of operational conditions for dehydration and purification experiments.

Mixture	Temperature (°C)	Membrane	Time (min)
Acetone/Water	30	M1	295
Acetone/Water	45	M1	90
Acetone/Water	30	M2	150
Acetone/Water/MeOH	50	M1	120
Acetone/Water/DCM	50	M1	145
Acetone/Water/MeOH/DCM	50	M1	120
MEK/Water/MeOH/DCM	50	M1	180
MEK/Water/MeOH/DCM/Acetone/Toluene	50	M1	210

**Table 3:** Summary of pure flux and permeance through M1 and M2.

Compound	Membrane	Temperature	Flux	Permeance (gpu)
		(°C)	(kg·h^−1^·m^−2^)	
Water	M1	30	1.01	11,950
	M1	45	2.63	13,019
	M2	30	1.39	16,866
Ethanol	M1	30	0.71	1262
	M2	30	0.47	836
Acetone	M1	30	0.76	291
	M2	30	0.49	188
MEK	M1	30	0.27	212
	M2	30	-	-

**Table 4:** Water permeance found in the literature and this study in the case of acetone dehydration.

Membrane	Temperature (°C)	Water Permeance (GPU)	References
PVA-MWCNT/CS	30	1910	[43]
crosslinked PVA-MWCNT/CS	30	1100	[43]
crosslinked PVA-MWCNT/CS	45	300	[43]
Pervap^TM^ 1210 (PVA/PAN)	50	3319	[44]
Pervap^TM^ 1210 (PVA/PAN)	60	4150	[44]
BTESE hybrid silica	30	5488	This study
BTESE hybrid silica	45	4220	This study
Zr doped BTESM hybrid silica	30	13,770	This study
